# A retrospective review of treatment patterns of antiemetic agents for chemotherapy-induced nausea and vomiting

**DOI:** 10.1177/2050312118767234

**Published:** 2018-05-01

**Authors:** Abdulrahman Alamri, Yousef A Alawlah, Yanru Qiao, Junling Wang

**Affiliations:** 1 PGY1 Pharmacy Practice Residency, Baylor St. Luke’s Medical Center, Houston, TX, USA; 2 Department of Pharmacy Practice, King Saud bin Abdulaziz University for Health Sciences, Riyadh, Saudi Arabia; 3 Department of Clinical Pharmacy and Translational Science, College of Pharmacy, The University of Tennessee Health Science Center, Memphis, TN, USA

**Keywords:** Chemotherapy, vomiting, nausea, oncology pharmacists, antiemetics

## Abstract

**Objectives:**

To evaluate the treatment pattern of antiemetic agents used for chemotherapy-induced nausea and vomiting in a tertiary hospital in Saudi Arabia.

**Methods:**

Over a period of 7 weeks, all new chemotherapy order sheets were collected and evaluated for chemotherapy-induced nausea and vomiting management. We compared each antiemetic regimen used for chemotherapy-induced nausea and vomiting prophylaxis with three international antiemetic guidelines by the following organizations: the Multinational Association of Supportive Care in Cancer, the American Society of Clinical Oncology, and the National Comprehensive Cancer Network for the clinician.

**Results:**

A total of 152 cancer patients were included in the study, for whom 289 chemotherapy physician orders included antiemetic regimens. Approximately 17.3% of the chemotherapy protocols had total minimal emetogenicity risk, 22.5% had low risk, 37.02% had moderate risk, and 23.18% had high risk. For acute emesis, 27.57% of the antiemetic regimens followed at least one of the three reference guidelines. For delayed emesis, only 20.16% of the antiemetic regimens adhered to at least one of the three reference guidelines.

**Conclusion:**

Adherence to treatment recommendations and antiemetics prescribing for chemotherapy-induced nausea and vomiting was suboptimal at this hospital. However, institutional antiemetic guidelines and oncology pharmacists could play an important role in better assessment and management of chemotherapy-induced nausea and vomiting.

## Introduction

Nausea and vomiting are considered two of the most common side effects/toxicities of chemotherapy in cancer patients. These side effects/toxicities may negatively affect the cancer patient’s quality of life or even lead to treatment withdrawal. Many complications including fluid and electrolyte disturbances, physical damage such as the Mallory–Weiss tear of the esophagus and dehydration could also result from chemotherapy-induced nausea and vomiting (CINV).^1–3^ Furthermore, nausea and vomiting in cancer patients could severely affect the nutritional status of the patients, requiring a decrease in the dosage of chemotherapy or further leading to an inability to tolerate future cycles of chemotherapy.^4^ CINV does not only happen to patients in hospitals but patients who receive chemotherapy in oncology clinics in an outpatient setting may also suffer from CINV, which may interfere with their daily functioning. The interference in patients’ daily life may be increased when CINV is severe or lasts for a long time. In addition, cancer patients rated nausea and vomiting as the most significant adverse events resulted from chemotherapy.^5^

CINV could occur as an acute or delayed onset. The acute onset of CINV generally occurs within 1 day of the initiation of chemotherapy and most of the time begin within 2 h and reach the maximum in 4–6 h after chemotherapy administration.^2,3^ The delayed onset of CINV occurs within 1–5 days after the initiation of chemotherapy, usually with the administration of cisplatin, and could occur with carboplatin, oxaliplatin, doxorubicin, and cyclophosphamide. The incidence of delayed emesis may be less frequent than acute emesis. However, it can be much harder to control with current antiemetic medications.^5^ In addition, some patients may experience anticipatory nausea or vomiting. Anticipatory emesis occurs few days before the next chemotherapy cycle with cancer patients who have experienced significant nausea and vomiting during previous cycles of chemotherapy. A proper control of CINV during the first cycle of chemotherapy would minimize the occurrence of anticipatory emesis.^6^

The management of CINV depends on several factors including emetogenic potential of chemotherapy agents, selection of proper antiemetic treatment, and specific patient risk factors.^1^ The emetogenic potentials of the chemotherapy regimen are classified into four levels according to the associated risk/probability of emesis: level 1 or minimal emetic potential with risk less than 10%, level 2 or low emetic potential with risk of emesis between 10% and 29%, level 3 or moderate emetic potential with risk of emesis between 30% and 89%, and level 4 or high emetic potential with risk/probability of emesis ≥90%.^7–9^ Different classes of antiemetic medications are available as antiemetic agents or regimen, mainly including neurokinin 1 antagonist, 5-hydroxytryptamine (HT)-3 receptor antagonists, and corticosteroids. These medications can be used alone or in combination, depending on the level of emetogenic potential of the chemotherapy agents. Other medication classes, including benzodiazepines, dopamine receptor antagonists, and cannabinoids, could offer additional protective benefit.^10,11^

The most important predictive factor for CINV is the emetogenicity of the chemotherapy agent and its route of administration. Other factors include young age, female gender, non-drinkers, nausea and vomiting during pregnancy or motion sickness, and rapid metabolizers of certain 5-HT3 receptor antagonists.^1,12,13^ Although the prevention of CINV can improve the tolerability of chemotherapy and increase compliance to cancer treatment, the management of CINV has been overall suboptimal. For example, Fabi et al. conducted an observational study to investigate treatment of delayed onset of CINV. A total of 149 patients were observed for one to four chemotherapy cycles. Among patients who received high or moderate emetogenic chemotherapy regimen, the proportions of appropriate treatment for delayed-type vomiting were only 43% and 64%, respectively. For patients who received a low emetogenic regimen, 68.5% received unnecessary prophylaxis.^14^ Another study by Patil et al.^15^ had similar findings.

Several barriers could influence physician attitudes to guideline adherence. These include patients’ medical condition, unawareness of the guidelines, lack of knowledge, and unfamiliarity or disagreement with specific guidelines.^16^ However, it has not been determined whether the aforementioned suboptimal treatment of CINV is common in Saudi Arabia. Studies on these patterns can help to provide evidence base for decision makers to understand the importance of addressing such issues. The objective of this study was to evaluate the treatment pattern of antiemetic agents used for CINV in a tertiary hospital in Saudi Arabia.

## Methods

### Study design

This prospective observational study was conducted in a large tertiary hospital with 800 beds in Saudi Arabia. The study was carried out in a 7-week period from 1 March 2008 to 25 April 2008.

### Patients

All adult (≥18 years) hematology–oncology patients admitted to the hematology/oncology department/clinic either in inpatients or outpatients setting to receive intravenous/oral chemotherapy were eligible for inclusion in the study. We excluded patients receiving intrathecal chemotherapy because of the low risk of inducing nausea and vomiting, patients receiving intravenous rituximab because of its minimal emetogenicity, non-oncology patients receiving chemotherapy for homogeneity of the study population, patients receiving radiation therapy because of possible contribution in the emetogenicity potential of radiation therapy agents, and pediatric patients because this study focused on adult population for population homogeneity.

### Data collection and analysis

A data collection sheet was developed to collect and record patient information such as age, gender, diagnosis, chemotherapy protocol, antiemetic regimen administered, and the emetic risk of each chemotherapeutic agent used in the protocol as well as the cumulative emetic risk for each chemotherapy protocol. A modified Hesketh’s method was used to calculate the cumulative emetogenicity risk.^13^ Hesketh’s method classified chemotherapy agent’s risk/probability of emesis to five levels: ≥90% (level 5), 60%–89% (level 4), 30%–59% (level 3), 10%–29% (level 2), and <10% (level 1).

The main study endpoint was whether an order matched the emetogenic risk of chemotherapy protocol regarding the acute and delayed onset of nausea and vomiting. This was determined based on CINV treatment guidelines.^7–9^ Simple statistical analysis and Microsoft Excel program were used in the analysis and the results are presented as mean, percentage, and frequency. The costs of the antiemetic regimens used for CINV prophylaxis in all physicians’ orders and the costs of the antiemetic regimens recommended in the three references guidelines were also compared. We estimate the cost of each antiemetic regimen used for CINV prophylaxis as the summation of the costs of actual antiemetic agents and the costs of the preparation and administration of the medication. The Center of Pharmaco-economics at the hospital estimated the cost of preparing and administering the medication based on the history of the cost of these services. This was estimated at US$1.33 for each intravenous push dose and US$0.45 for each oral dose.

According to sample size calculation method for determining a sample proportion, a maximum sample size of 131 is required for a proportion of 40%–60%, a confidence interval width of 12%, and a significance level of 0.05.^14,15^ Ethical approval for this study was deemed exempt by National Guard Health Affairs Research Committee, Saudi Arabia. Patient consent is not applicable since only existing medical records were reviewed.

## Results

A total of 152 cancer patients were included in the study, for whom 289 chemotherapy physician orders were collected. For some patients, there were more than one physician orders for different chemotherapy cycles. The complete demographic characteristics were summarized (Table 1). The mean age was 46.5 years (standard deviation: 13.7). The ratio of men to women was 1:1.5 and the ratio of physicians’ order for men to women patients was 1:1.2. Approximately 80% of the chemotherapy orders were in outpatients setting and 67.1% of the orders were for oncology patients. Breast and colon cancers were the two most common diagnoses.

**Table 1.** Demographic characteristics of the study population.

Variables	Number (%)
Total patients	152
Total physician orders	289
Age (mean ± standard deviation)	46.5 ± 13.7
Gender	
Male	62 (40.8)
Female	90 (59.2)
Physician orders	
Male	129 (44.6)
Female	160 (55.3)
Setting	
Outpatient	231 (79.9)
Inpatient	58 (20.1)
Type of physicians’ order	
Oncology physicians’ order	194 (67.1)
Hematology physicians’ order	95 (32.9)
The most common diagnosis	
Breast cancer	94 (32.5)
Colon cancer	77 (26.6)

Most of the patients received chemotherapy regimens that were deemed as with moderate emetogenicity risk. For acute emesis prophylaxis, 27.6% of the antiemetic regimen orders followed at least one of the three guidelines. For delayed emesis prophylaxis, only 20.2% of the antiemetic regimen orders adhered to at least one of the three guidelines. Overall, 13.5% of the chemotherapy protocols were optimal regarding CINV prophylaxis in following at least one of the three guidelines for acute and delayed CINV, 46.5% of the chemotherapy protocols were over-prophylaxis with antiemetic medications, and 40.0% of the chemotherapy protocols were under-prophylaxis (Table 2).

**Table 2.** Percentage of adherence to treatment guidelines for chemotherapy-induced nausea and vomiting (CINV) prophylaxis.

Variables	Percentage of adherence
Types of emesis	
Acute emesis	27.6
Delayed emesis	20.2
Status of adherence	
Optimal	13.5
Over-prophylaxis	46.5
Under-prophylaxis	40.0

Some specific medication utilization issues were identified (Table 3). The first and the most frequent issue was that intravenous (i.v.) granisetron and dexamethasone were over-utilized for acute emesis prophylaxis, while the more economical oral granisetron and dexamethasone were under-utilized in the current practice. Intravenous dosage forms were used in all the orders containing granisetron and dexamethasone for acute emesis prophylaxis. The second common issue was the over-utilization of metoclopramide for chemotherapy protocols with moderate or high emetogenicity, which occurred in 60.6% of orders. Metoclopramide was indicated for emesis prophylaxis of low emetogenicity drugs.^8^ The third issue was the inappropriate use of expensive medications such as granisetron for acute emesis prophylaxis. In 27.2% of the orders containing chemotherapy protocols with low emetogenicity risk, granisetron was utilized. The guidelines, however, recommended granisetron for chemotherapy agents with a high or moderate emetogenicity risk only.^7–9^ The fourth issue was the under-utilization of the aprepitant which was only used in 12.2% of the time when indicated. Aprepitant is neurokinin 1 antagonist approved by the Food and Drug Administration (FDA) for CINV prophylaxis for chemotherapy protocol with high emetogenic risk.^7–9^

**Table 3.** Some specific medication utilization issues.

Specific medication utilization issues
1. Over-utilization of intravenous granisetron and dexamethasone for acute emesis prophylaxis
2. Over-utilization of metoclopramide for chemotherapy protocols with moderate or high emetogenicity
3. Inappropriate use of expensive medications such as granisetron for acute emesis prophylaxis
4. Under-utilization of aprepitant
5. Improper assessment of emetogenicity risk
6. Most antiemetic regimens prescribed in fixed-dose combinations and regimens despite different emetogenic risks of each protocol

The fifth issue was that risk of emetogenicity was not assessed properly. For example, patients receiving high emetogenicity chemotherapy protocol were treated with granisetron and dexamethasone only, although the use of aprepitant, granisetron, and dexamethasone ± lorazepam was indicated.^7–9^ The last issue identified was that most antiemetic regimens were prescribed in fixed-dose combinations and regimens despite different emetogenic risks of each protocol. For example, fixed-dose combinations of granisetron and dexamethasone were used frequently by some prescribers for both protocols with high emetogenic risk and those with low emetogenic risk while failing to adjust the dosage according to the level of emetogenicity. The cost savings per year using effective antiemetic guidelines for CINV prophylaxis were estimated to be approximately US$14,450.

## Discussion

Our study evaluated the prescribing practice of antiemetic medications for CINV prophylaxis in a large tertiary hospital in Saudi Arabia. The results showed that physicians were frequently not following at least one of the three well-known guidelines. We also identified some specific medication utilization issues. We found that lower than 14% of the antiemetic regimens were considered optimal for prophylaxis of both acute and delayed onset compared to all three standard guidelines, lower than 50% of antiemetic regimens were over-prophylaxis, and over 40% were under-prophylaxis. Although this study used simple description of data, it documented an important gap in adherence to treatment recommendations and antiemetic guidelines.

Many previous studies addressed the importance and the benefits of following antiemetic guidelines to optimize CINV management. A multicenter prospective study was conducted in eight European countries including 991, 888, and 769 patients who completed the first three cycles of chemotherapy. Patients received highly and moderately emetogenic chemotherapy agents. That study evaluated the predictors for CINV development, including patient- and treatment-related characteristics.^17^ One of the most important predictors was the use of antiemetic medications inconsistent with international guidelines. The study showed that the adherent to antiemetic guidelines was critical to optimizing CINV management.^17^ Another small retrospective cohort study included 49 pediatric oncology patients who completed their first cycle of chemotherapy. That study found that 14 out of 49 patients experienced breakthrough nausea and vomiting. All antiemetic regimens that were ineffective to prevent CINV were found to be inconsistent with the standard guidelines.^18^

The under-utilization of aprepitant, an FDA-approved agent, is an important issue. Aprepitant is recommended to be used with 5-HT3 receptor antagonists in all three reference guidelines for CINV prophylaxis for high emetogenic chemotherapy protocol.^7–9^ In comparison between usage of intravenous ondansetron and oral dexamethasone plus aprepitant versus intravenous ondansetron and oral dexamethasone plus placebo, three randomized, double-blind, placebo-controlled trials showed that overall complete control of CINV was achieved more in patients receiving intravenous ondansetron and oral dexamethasone plus aprepitant than the comparison group (63%–73% versus 43%–52%, *p* < 0.01 for all comparisons).^19^

This study found that most of the antiemetic regimens were ordered in fixed-dose combinations, although doses of some antiemetic agents should have been adjusted according to the level of emetogenicity. This could be related to the difficulty in assessing the emetogenicity risk and selecting the right dosage of antiemetic agents without referring to proper guidelines. In this study, it was estimated that following the antiemetic guidelines in prescribing CINV prophylaxis could have saved approximately US$14,450. The saved money could be a great resource to help in other supportive care and may be utilized for other patient care services.

Regarding the CINV management, pharmacy staff at the hospital may be able to play an important role in helping physicians to calculate the emetogenicity risk for each chemotherapy protocol, select the appropriate antiemetic regimen, and review the chemotherapy orders as well as the antiemetic regimens. Previous studies identified the role of the pharmacist with cancer patients. Oncology pharmacist can be of great benefit in many areas, including but not limited to management of complications resulting from chemotherapy treatment, chemotherapy counseling services, thrombosis risk management, pain management, drug interactions, and development of guidelines for management of CINV. The pharmacist has the advantage of being the expert of drug therapies, adverse drug reactions, and drugs monitoring.^20–24^

The lack of guidance for assessment of the emetogenic risk of chemotherapy protocols may have contributed to the inconsistency between the clinical practice in this hospital and the standard guidelines. Therefore, as a follow-up of this study, an institutional CINV prophylaxis guideline was developed at the study institution. The head of the hematology and oncology department in the hospital reviewed and approved these guidelines. Subsequently, it was implemented after being reviewed by the Pharmacy and Therapeutics (P&T) committee for feedback and receiving final approval. The guidelines also stipulated that no prescription orders could be dispensed unless deemed to be consistent with the institutional CINV management guidelines. These measures were able to minimize the underestimation and overestimation of the emetogenic risk of chemotherapy protocols and help the prescribers to order appropriate antiemetic regimens. Currently, the consistency between the prescribing pattern of the CINV prophylaxis and the hospital’s CINV management guidelines has been improved to almost 100%.

This study made a significant contribution to the existing literature by identifying issues in prescribing antiemetics for CINV. However, this study also has limitations. The practice patterns in only one hospital were studied, so the study generalizability may be limited. Furthermore, this study did not examine the impact of implementation of developed guidelines on patient outcomes in a comprehensive manner.

## Conclusion

The adherence to treatment guidelines of antiemetics prescribing for CINV was suboptimal in this tertiary hospital in Saudi Arabia. Institutional management guidelines and the help from oncology pharmacists could facilitate better assessment and management of CINV. Future studies should examine the generalizability of the study findings.
